# Explaining the ranchers’ behavior of rangeland conservation in western Iran

**DOI:** 10.3389/fpsyg.2022.1090723

**Published:** 2023-01-04

**Authors:** Moslem Savari

**Affiliations:** Department of Agricultural Extension and Education, Agricultural Sciences and Natural Resources University of Khuzestan, Mollasani, Iran

**Keywords:** rangeland protection, natural resources, livestock and rangeland balance, value-belief-norm, ranchers’, Iran

## Abstract

Rangeland are one of the most important natural habitats for the protection of living and non-living organisms. Degradation of rangeland is one of the biggest threats to biodiversity loss. Ranchers’, as the most important key stakeholders who have a direct relationship with rangelands, have put a lot of pressure on the rangeland sector in recent years, so that this natural habitat has been destroyed. Therefore, existing rangelands must be preserved to protect biodiversity. One of the most important strategies to protect rangeland is to change the behavior of ranchers to friendly and ecological behaviors in the environment. In this regard, this study was conducted with the general aim of explaining the ranchers’ behavior of rangeland conservation in western Iran. The main research tool was a questionnaire whose validity was confirmed by a panel of experts and its reliability was confirmed by Cronbach’s alpha coefficient. In this study, value-belief-norm (VBN) was used to identify research variables. The results showed that ranchers have used different behaviors to protect rangelands and egoistic behaviors are the main cause of the destruction of this natural habitat. In addition, the results of this study showed that the framework used was an efficient theory because it explains 53.9% of rangeland conservation behavior. Finally, in this study, based on the research findings, applied policies for the protection of natural rangelands were presented so that in addition to rangeland conservation, biodiversity can also be preserved.

## Introduction

Nearly half of human societies depend on the products and services of the rangeland, the largest ecosystem on Earth (Eddy et al., 2017; Brunson et al., 2021). It is estimated that 30–50% of the world’s land surface is covered by rangelands (Sugita et al., 2007; Dinan et al., 2021). In Iran, there are about 84.7 million hectares of grazingland, which accounts for 52% of the country’s total land surface. The total grazing area in Iran consisted of 8.5, 25.3, and 66.2% of dense, semi-dense, and low-density pasture, respectively (Asaadi and Yazdi, 2011; Karimi and Saghaleini, 2021). Rangeland is considered one of the most important sources of production in this country and plays a key role in supplying forage for domestic animals and food production (Karimi and Saghaleini, 2021; Savari and Zhoolideh, 2021; Savari and Moradi, 2022). Grazing land is therefore one of the most important natural resources of a country, providing the basis for other activities such as agriculture and animal husbandry. Hence, taking measurements to conserve these natural resources is of high importance (Noguera-Mendez et al., 2016). Rangeland ecosystems provide several key services, including medicinal plant production, forage provision, climate regulation, soil and water conservation, and environmental conservation (Prager et al., 2015). In recent decades, many efforts have been made to protect rangelands. For instance, the Returning Grazing Land to Grassland project was launched in 2003 (Wang et al., 2013). Nevertheless, these efforts have not been effective as the degradation of rangelands is increasing (State Forestry Administration [SFA], 2005, 2011, 2015). In the last three decades, Iranian rangelands have been destroyed to a greater extent than those of European and American countries, so that more than 20% have been destroyed quantitatively and qualitatively (Food and Agriculture Organization [FAO], 2013). There are 124 million livestock units in Iran, 83 million of which depend on Iranian rangelands. However, Iranian rangelands are only capable of feeding about 37 million livestock units within 7 months or about 24.6 million livestock units within a year. Consequently, Iranian rangelands are currently being used 2.2 times more than their allowable capacity (Karami et al., 2021). As a result, the imbalance between the livestock population and the tolerance capacity of Iranian rangelands has destroyed many grazing areas and subsequently eroded the soils (Savari et al., 2022a,b). Therefore, illegal grazing is the main cause of rangeland degradation (Noguera-Mendez et al., 2016; Wassie, 2020; Besada, 2021) because livestock grazing affects various components of rangeland ecosystems such as soil, water, floral composition, diversity, and forage quality on the performance of the whole ecosystem (Hein, 2006; Retzer, 2006; Eldridge et al., 2016; Lu et al., 2017; Karami et al., 2021). Intermittent grazing, on the other hand, is associated with a positive effect on plant traits and rangeland ecosystems. Seasonal grazing restricts the selection of grazing livestock, which reduces the production of invasive seeds and increases flowering traits such as plant growth and survival (Earl and Jones, 1996; Zhang et al., 2017). However, more than 70% of rangeland degradation is attributed to human activities, to improve rangeland conservation, ranchers in particular need to improve their environmental behavior (Food and Agriculture Organization [FAO], 2013; Lepak et al., 2021). According to this evidence, ranchers are more involved in the rangeland ecosystem than any other part of society and play a key role in its degradation and conservation (Folke, 2006; Kovács et al., 2021). Currently, pastoralist behavior in rangeland conservation is not well-studied (Lu et al., 2017; Ohta et al., 2020; Karami et al., 2021; Karimi and Saghaleini, 2021). What has been emphasized more in past studies has been the control of livestock farmers’ behavior through incentives and restrictions (Katuwal, 2012; Kovács et al., 2021) and policy makers have planned without considering the viewpoints and attitudes of farmers. Meanwhile, examining people’s views and norms precedes any activity in the field of environmental protection (Savari et al., 2022b).

The use of social-psychological models and theories is one of the most important methods for studying conservation behavior and its preventive factors (Turaga et al., 2010; Yazdanpanah et al., 2015; Savari and Gharechaee, 2020; Karimi and Saghaleini, 2021; Savari et al., 2022c). Environmental psychological studies are important because they show that sustainable behavior in the environment requires individuals to internalize norms and behaviors, as incentives and constraints cannot produce sustainable manners in them (Savari et al., 2022d). According to psychological theories, the pro-environmentally behavior of people is influenced by psychological factors such as attitudes, beliefs, feelings, norms, and values (Bagheri et al., 2019; Rezaei et al., 2019; Savari and Gharechaee, 2020). However, sociologists and psychologists have proposed various theories to study the environmental behavior of users, the most important of which are the following norm activation model (NAM) (Schwartz, 1977), theory of planned behavior (TPB) (Ajzen, 1991), and value-belief-norm (VBN) (Stern, 2000). Among these views, VBN received attraction due to its comprehensiveness, simplicity of variable measurement, emphasis on psychological aspects, especially internal values and beliefs, and relevance of variables to environmental factors (Chen, 2015; Lind et al., 2015; Veisi et al., 2020). Interestingly, only this theory considers the worldview of human ecology in nature (Chen, 2015). Thus, this study aimed to identify the factors that influence ranchers’ behavior of rangeland conservation in western Iran. Two main objectives were pursued: (i) to use VBN ability in identifying the factors influencing rangeland protection behaviors, and (ii) to develop practical interventions to strengthen rangeland protection behaviors in Iran.

## Theoretical framework

### Value-belief-norm (VBN)

There are several approaches to explain human behavior in the use of natural resources such as water, forests, and rangelands. These methods are called logical and moral strategies with different assumptions (Steg and Vlek, 2009). Stern et al. (1999) combined values theory, including the New Ecological Paradigm (NEP) and NAM, and developed VBN theory to describe environmental behaviors. As a theory, VBN examines normative factors that contribute to sustainable attitudes and behaviors (Fornara et al., 2016). VBN theory provides a causal chain of explanations for environmental behavior (Chou, 2014; Huffman et al., 2014; Stevenson et al., 2014) that moves from stabilized and permanent elements of personality and belief to a greater focus on the unfortunate consequences of one’s values and personal responsibility to reduce risk (Chen, 2015; Lind et al., 2015).

As an moral approach, NEP emphasizes moderation and balance in the exploitation of nature because of the limitations it places on man’s use of natural resources. In other words, its strategy considers humans as part of nature, in contrast to the prevailing paradigm, which is a rational approach (Dunlap et al., 2000). According to VBN theory, this view introduces different values for natural resources such as forests, rangelands, and water that determine the underlying attitude and behavioral framework of individuals toward the environment (Ibtissem, 2010). VBN assumes that personal moral norms are activated by individuals who become aware of the adverse impacts of certain environmental conditions that threaten their desired values. As a result, individuals take responsibility for these devastating consequences (Stern, 2000; Zhang et al., 2020).

**FIGURE 1:**
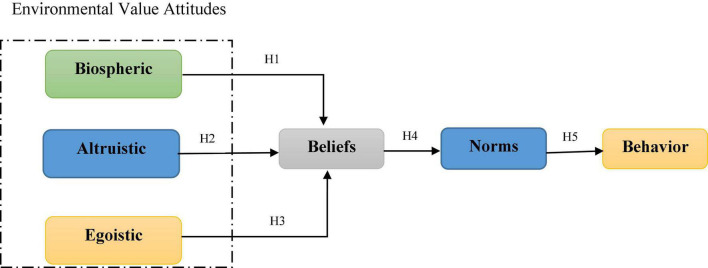
Causal chain of variables in VBN theory (Stern, 2000).

The theory considers the variables of environmental values attitudes (altruistic, biospheric, and egoistic), beliefs, norms, and behaviors that have specific relationships (Stern, 2000; Bijani and Hayati, 2013). According to VBN theory, behavioral beliefs are based on biospheric, altruistic, and egoistic values (Stern, 2000; Chen, 2015). Altruistic and biospheric orientations positively influence behavioral beliefs, whereas egoistic orientations negatively influence them (Hiratsuka et al., 2018; Zhang et al., 2020). Egoistic people tend to evaluate environmental aspects based on how the environment affects them. In other words: If they understand that there is a cost to using protective behaviors in the environment, they should avoid them (Chen, 2015). Those who are altruistic, on the other hand, typically evaluate aspects of the natural environment based on benefits and costs to human groups (Cho et al., 2013). Biospheric people evaluate the environment based on the benefits and costs it has to the ecosystem. People who value natural resources and the environment are more likely to prevent threatening situations to the ecosystem (Chen and Chai, 2010). In other words, people’s values influence their perceptions of the consequences of ecosystem change for themselves, for other people, and other species within the ecosystem (Stern and Dietz, 1994). The term environmental beliefs refer to a system of attitudes that determine an individual’s norms toward the environment and are the guiding principles in interacting with the environment (Corral-Verdugo et al., 2003). People’s beliefs and attitudes are the first things that affect the environment and determine their behavior toward it, as they specify how the different components of the ecosystem are valued (Wensing et al., 2019; Ranjbar and Naeimi, 2020). Consequently, understanding people’s environmental beliefs and identifying the factors that influence them is critical to environmental behavior research (Budak et al., 2005). According to VBN theory, personal norms are a key factor that directly influences environmental behavior (Stern, 2000; Phipps et al., 2013; Chou, 2014). In fact, moral norms are an internal emotional concept that ethically compels one to take actions that are consistent with one’s values (Karimi and Saghaleini, 2021). Moral norms refer to one’s sense of moral commitment to do or refrain from doing certain things that lead to environmentally friendly behavior (Yuan et al., 2021). Finally, pro-environmental behavior in this theory refers to conscious actions of individuals toward the environment, which include a wide range of feelings, desires, and willingness to perform desired actions. These behaviors aim to minimize the negative impact of human activities on the environment and improve it (Savari et al., 2021). Therefore, research hypotheses were raised based on Figure 1:

H1. Environmental value attitudes (Biospheric) has a positive and significant effect on beliefs.

H2. Environmental value attitudes (Altruistic) has a positive and significant effect on beliefs.

H3. Environmental value attitudes (Egoistic) has a negative and significant effect on beliefs.

H4: Beliefs has a positive and significant effect on norms.

H5. Norms has a positive and significant effect on rangeland protection behavior.

## Materials and methods

### Statistical population and sampling method

The statistical population of the study included all ranchers in the Saral district of Kurdistan province (Western Iran). According to Krejcie and Morgan (1970), 385 pastoralists in 15 villages were selected for the study. The sampling was conducted as a multistage stratified method with proportional allocation. This means, we tried to select the samples with proper distribution at the regional level so that the selected samples have high capability and reliability.

### Study area

**FIGURE 2:**
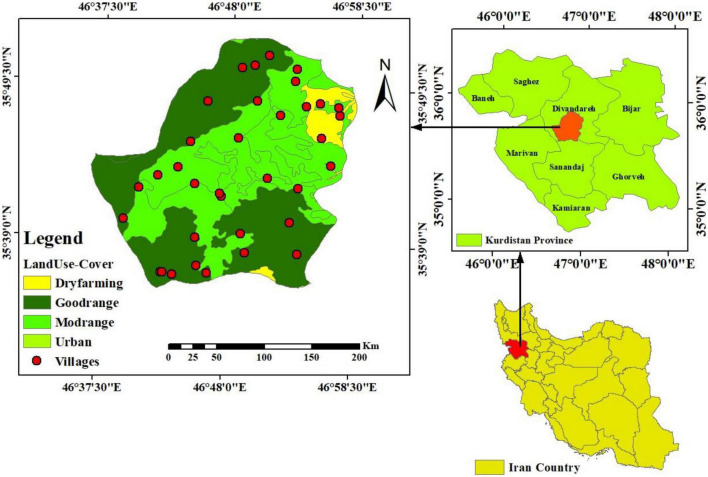
Study area.

This investigation was carried out in 2020 and 2021 in a mountainous area approximately 35 km from Sanandaj (46450–46490 E and 35320 N–35360 N latitude) at an altitude of 2145 m (Figure 2). Saral district with an area of 1000 km^2^ is located in Kurdistan province in western Iran. The average annual rainfall in this region is 480 mm (Iran Meteorological Organization, 2019). The climate in this area is cold and semi-arid. Saral has sandy loam soils which are excellent for grazing livestock (cattle, sheep, goats). Grazing (grazed sites) in this area by sheep, goats, cows and wild animals takes place from late April to late July without any additional management practices (Karami et al., 2021). Ecologically, Saral district is a safe habitat for diversity of plants and animals. Notably, this natural region has not been studied in an accurate and systematic way so far, and the majority of studies have focused on the plant and animal species in this region rather than the factors leading to degradation. The main occupation of the people in the Saral region is animal husbandry, as it is one of the most vulnerable areas for animal husbandry and is known as such throughout Iran (Figure 2).

### Participants

According to the results, the average age of the participants was 52.44 ± 7.33 years. In addition, the results showed that the majority of the respondents 274 (71.16%) were male and 345 (89.61%) were married. In terms of education, results showed that among the respondents 73 were illiterate, 85 were primary school students, 82 were middle school students, 79 were graduate diplomas, and 66 received higher education. In addition, the average number of livestock was 27.22 sheep, 9.12 goats, and 1.24 cows. In addition, the livestock ranchers obtained more than 50% of the feed required by their domesticated animals from the rangelands and the animals grazed on the rangelands for more than 180 days.

### Measurements

The main research tool was a questionnaire consisting of two general parts. The first part dealt with the personal and occupational characteristics of pastoralists. The second part consisted of 33 items measuring theoretical structures (VBNs) divided into six sections: (i) five items measuring biospheric value attitude, (ii) five items measuring altruistic value attitude, (iii) five items measuring evaluation of egoistic value attitude, (iv) five items measuring moral norms, (v) five items measuring behavioral beliefs, and (vi) eight items measuring rangeland conservation behaviors (RCB). Then, participants were asked to express their agreement or disagreement on the Likert scale (1-very low to 5-very high) to reduce statistical errors (Fornell, 1992). One of the most important points of this research was to measure the theoretical variables (VBN) using previous studies. The items of the questionnaire are shown in Table 1.

**TABLE 1:** Research measurement concepts and variables.

Construct	Measurement items	References
Biospheric	In addition to humans, plants, and animal species must also use the rangelands.	Dunlap et al., 2000; Chen and Chai, 2010; Hiratsuka et al., 2018; Veisi et al., 2020
	Livestock ranchers must also respect the rights of other plant and animal species.	
	I think we should behave fairly in nature.	
	Conserving the environment and rangelands takes precedence over livestock development.	
	Preserving vegetation and rangelands is more vital than forage supply for ranchers.	
Altrisitic	In my opinion, pasture conservation is a priority for ranchers.	Bijani and Hayati, 2011; Cho et al., 2013; Fornara et al., 2016; Zhang et al., 2020
	No rancher is allowed to destroy rangelands due to increase production (meat and milk), because it means losing the rights of other ranchers.	
	No rancher should leave his animals in the wilderness when it rains, because rangelands belong to all ranchers.	
	Grazing land belongs to all generations and we should not destroy it.	
	Since all ranchers have equal rights to the rangelands, no rancher can let as many cattle graze there as they want.	
Egoistic	Because of today’s economic conditions, I cannot think of conserving the rangelands and their collective interests.	Bijani and Hayati, 2011; Chen, 2015; Hiratsuka et al., 2018; Veisi et al., 2020
	For me, increasing my livestock and income is more important than preserving rangelands.	
	Making more money is of greater importance than conserving rangelands.	
	I am allowed to use the rangelands as much as I want.	
	Providing forage for my livestock is more important than other ranchers.	
Moral norm toward pasture conservation	I think maintaining the rangelands is a moral obligation.	Stern, 2000; Savari et al., 2021
	I believe that the conservation of rangelands should not be neglected.	
	If I fail to protect rangelands, I feel guilty.	
	It is very important to me that ranchers protect their rangelands.	
	Ranchers help each other protect rangelands and interact well together.	
Belief in pasture conservation	I believe that rangelands are less abundant than in the past and we need to conserve them	Stern, 2000; Chen, 2015; Zhang et al., 2020
	I believe that overgrazing can destroy rangelands	
	I believe that rangelands do not have as much livestock capacity as in the past and we need to reduce the number of livestock	
	I believe that ranchers should not overuse grazing land to gain forage.	
	I believe that ranchers should avoid fixed grazing on rangelands.	
Rangeland conservation behaviors (RCB)	Reduction of livestock in grazing areas (balance between livestock and rangelands).	Karimi et al., 2021; Karimi and Saghaleini, 2021
	Purchase of fodder from other places to reduce rangeland on rangelands	
	Use of rotational grazing instead of fixed grazing on rangelands.	
	Cultivation of compatible native plants in grazing areas	
	Avoid heavy grazing on rangelands	
	Prevent flowering and seed production of invasive and inappropriate grazing plants	
	Prevent livestock from entering rangelands too early in the spring.	
	Assist with grazing exclusion programs and removal of livestock from rangelands	

### Validity and reliability of instrument

An expert panel reviewed the survey draft and questions before interviewing farmers to assess the general indicators measured. The panel included professors from the fields of agricultural extension and education, environment, psychology, social sciences, and agricultural sciences, and modifications were made based on their opinions until final approval. In addition, Cronbach’s alpha coefficients and combined reliability were calculated to test the reliability of the questionnaire, which showed acceptable values (Table 3).

### Data analysis

Data were collected and analyzed using SPSS_23_ and Smart Pls software. Structural Equation Modeling (SEM) is widely used by researchers because it provides the opportunity to test theories in the form of equations between variables. Moreover, this method can take into account the measurement error so that researchers can analyze the data by specifying the measurement error (Khoshmaram et al., 2020). Conventional models in SEM consist of two parts (Hair et al., 2014). A measurement model that examines how hidden variables are explained by explicit variables (questions), and a structural model that shows how hidden variables are related to each other (Hair et al., 2017). Furthermore, in this study, the ISDM1 index was applied to classify rangelands conservation behaviors (Gangadharappa et al., 2007):


(1) Low:A<Mean-1/2⁢Sd

(2) Medium:Mean-1/2⁢Sd<B<Mean+1/2⁢Sd

(3) High:C>Mean+1/2⁢Sd

## Results

### Assessing the status of VBN model variables among respondents

As explained in the research method, the ISDM index was used to group the variables applied to the VBN model and rangelands protection behavior among ranchers’. According to the obtained results, the studied ranchers’ did not show any considerable rangelands protection behavior, and only 24.94% had high protection behaviors, while the majority (48.57%) exhibited moderate rangelands protection behaviors. Moreover, two variables of VBN model, including moral norm and belief were at higher rank rather than other variables (Table 2).

**TABLE 2:** Grouping the situation of VBN model variables among studied woman.

Variable	Mean	SD	ISDM category					
			**Low**		**Medium**		**High**	
			**Frequency**	**Percent**	**Frequency**	**Percent**	**Frequency**	**Percent**
Behavior conservation	2.88	0.688	102	26.49	187	48.57	96	24.94
Biospheric	2.69	0.715	114	29.61	201	52.20	70	18.19
Altrisitic	2.74	0.608	107	27.97	198	51.42	80	20.61
Egoistic	2.96	0.705	98	24.45	214	55.58	73	19.97
Moral norm	3.08	0.711	87	22.59	189	49.09	109	28.32
Belief	3.24	0.639	80	20.77	185	48.05	124	31.18

**TABLE 3:** The results of fit of measurement models.

Constructs	Constructs	ƛ	*t*	Reliability and validity statistics
	BI1	0.833	28.241	AVE: 0.671
	BI2	0.882	39.714	CR: 0.919
Biospheric	BI3	0.876	29.404	α: 0.887
	BI4	0.829	20.929	
	BI5	0.683	12.483	
Altruistic	AL1	0.790	26.157	AVE: 0.573
	AL2	0.620	8.851	CR: 0.886
	AL3	0.679	10.816	α: 0.788
	AL4	0.794	17.730	
	AL5	0.781	14.690	
Egoistic	EG1	0.654	11.821	AVE: 0.589
	EG2	0.801	24.159	CR: 0.853
	EG3	0.766	16.159	α: 0.807
	EG4	0.797	23.611	
	EG5	0.736	14.750	
Beliefs	BE1	0.854	34.992	AVE: 0.618
	BE2	0.846	23.154	CR: 0.867
	BE3	0.762	18.529	α: 0.819
	BE4	0.769	15.835	
	BE5	0.609	10.481	
Norms	NO1	0.729	18.841	AVE: 0.527
	NO2	0.757	15.613	CR: 0.822
	NO3	0.765	17.147	α: 0.771
	NO4	0.675	11.170	
	NO5	0.704	13.811	
Behavior	BEH1	0.746	18.247	AVE: 0.577
	BEH2	0.840	26.908	CR: 0.903
	BEH3	0.802	17.216	α: 0.873
	BEH4	0.768	18.259	
	BEH5	0.651	13.403	
	BEH6	0.701	13.477	
	BEH7	0.672	12.596	
	BEH8	0.670	11.216	

### Measurement model evaluation

This step determines whether the structures were measured accurately. To answer this question, the three phases of unidimensionality, reliability and validity, and discriminant validity were examined.

#### Unidimensionality

This step was assessed by factor loading and *t*-values. According to the values presented (Table 3), it can be claimed that the factor loading values presented for selected markers were statistically significant (above 0.6) and at the one percent error level (*P* < 0.01). The results confirmed the unidimensionality of the selected markers. Therefore, it can be concluded that the markers were selected correctly for the evaluation of research structures that measure exactly the same component.

#### Reliability and validity

In this step, the Combined Reliability (CR), Cronbach’s alpha, and Average Variance Extracted (AVE) were examined. The results presented in Table 3 indicated that the CR of all the structures in the proposed research model was more than 0.60 and their Cronbach’s alpha coefficients were more than 0.70. Moreover, the AVE for all structures of the proposed research model was above 0.50. Therefore, all latent variables had high reliability and validity, meaning that the items measuring the research structures were carefully selected and allowed the experiment to be repeated.

#### Discriminant validity

Diagnostic validity exists when questions measuring one variable are distinct or distinguishable from questions measuring other variables. Based on statistics, the research variables are of adequate diagnostic validity if the root mean of the calculated AVE variance between them is greater than the correlation between them (Fornell and Larcker, 1981). According to the results presented in Table 4; it can be seen that the root mean of the extracted variance for the research structures (0.71 < AVE < 0.92) was more than the correlation between them (0.33 < r < 0.55). This result confirmed the diagnostic validity of the structures in the proposed research model.

**TABLE 4:** Correlations with square roots of the AVEs.

Constructs	1	2	3	4	5	6
1-Biospheric	0.92^a^					
2-Altruistic	0.45^**^	0.88^a^				
3-Egoistic	0.55^**^	0.41^**^	0.88^a^			
4-Beliefs	0.43^**^	0.33^**^	0.37^**^	0.78^a^		
5-Norms	0.39^**^	0.42^**^	0.51^**^	0.35^**^	0.84^a^	
6-Behavior	0.45^**^	0.41^**^	0.42^**^	0.38^**^	0.36^**^	0.71^a^

^a^The square roots of AVE estimate. **Correlation is significant at the <0.01 level.

#### Evaluation of the research structural model

Various indicators were used in evaluating the fit of the research structural model (Table 5). Based on the proposed values of the presented indicators and the reported values, it can be claimed that the model had a good fit and the research hypotheses could be tested based on the model.

**TABLE 5:** Summary of goodness of fit indices for the measurement model.

Fit index	SRMR	D-G1	D-G2	NFI	RMS-theta
Suggested value	<0.1	>0.05	>0.05	>0.90	≤0.12
Estimated value	0.07	0.517	0.254	0.97	0.07

**FIGURE 3:**
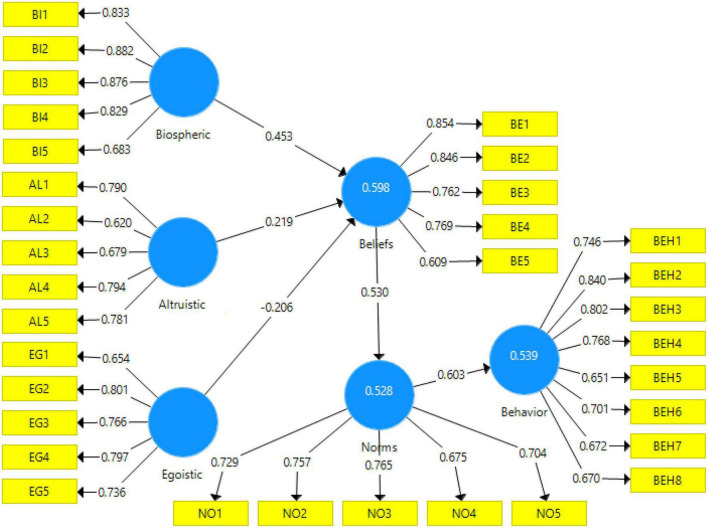
Path model with standardized factor loadings.

**FIGURE 4:**
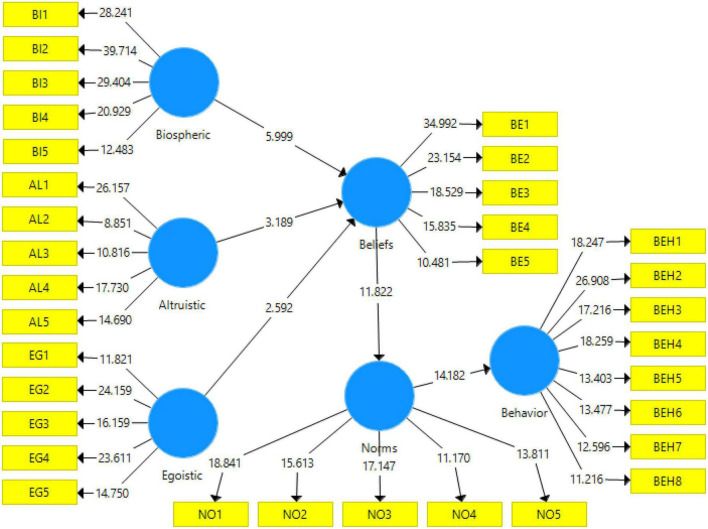
Path model with *t*-values.

The measurement and structural models of the research were confirmed by confirmatory factor analysis. Then, to test the hypotheses of the proposed conceptual model of the research, the method of path analysis (structural model evaluation) was used. The path model of the research was represented by showing standardized loadings of the factors and the *t*-values in Figures 3, 4.

#### Test of the research hypotheses

In this step, the results of the final effect of the variables on the use of RCB were presented. The bootstrapping method was used to evaluate the significance of the path coefficient or beta. We used bootstrapping on 100 and 300 samples. According to the results, the significance of the parameters did not change in both cases and the results were valid. This is because the sample size did not affect the significance of the relationships between the variables, except for the value of the t-statistic. Therefore, regression models could be used to test hypotheses. The results showed that all research hypotheses were confirmed based on the predicted relationships. In addition, the research variables were able to explain 53.9% of the variance of RCB (Table 6).

**TABLE 6:** Results of research hypotheses.

Hypothesis	ƛ	t	Result	VIF	R^2^	Q^2^
H1: Biospheric→Beliefs	0.453	5.999	Confirm	1.685	0.598	0.154
H2: Altruistic→Beliefs	0.219	3.189	Confirm	1.524		
H3: Egoistic→Beliefs	−0.206	2.592	Confirm	1.638		
H4: Beliefs→Norms	0.530	11.822	Confirm	1.770	0.528	
H5: Norms→Behavior	0.603	14.182	Confirm	1.331	0.539	

## Discussion

This study investigated the factors influencing the use of RCB among ranchers in western Iran using the VBN psychological model. In western Iran, this study represented the first attempt to protect rangelands, as past studies on pasture conservation have paid less attention to pastoralists who have a very close relationship with rangelands. Previous studies have focused on restrictions and incentives to examine ranchers’ behavior. Because there was a belief that rangelands can be preserved through proper grazing exclusion and demarcation, so psychological studies were rarely considered. Recently, more attention has been paid to environmental psychology because researchers believe that the application of constraints and incentives cannot produce sustainable behavior in the environment. When those motivations and restraints are removed, people’s behavior returns to its original state. As a result, there is now more focus on norms, attitudes, and beliefs of individuals that can influence sustainable behavior in the environment (Savari et al., 2022b). Since very little research has been conducted in this area, this study used VBN theory because this strategy contains value attitudes, beliefs, and moral norms in its structure to examine the factors that influence RCB. Since this study examined the various effective factors on RCB and provided new insights in this regard, its findings can fill the gap of previous research in the literature and assist countries involved in uncontrolled rangeland destruction. SEM was used to test the research hypotheses presented in the following order.

This study showed that VBN theory was an effective model to examine the factors affecting RCB, not only because it confirmed all predicted relationships, but also because it was able to accurately predict more than 50% of RCB use. In addition, the results illustrated that environmental value attitudes can have a significant impact on beliefs about rangeland conservation. Our result is consistent with (Stern, 2000; Chen, 2015; Lind et al., 2015; Veisi et al., 2020). This finding can be explained by the fact that value attitudes are constantly mentioned as determining human behavior in the environment. In other words, people’s behavior is influenced by their attitudes toward their natural environment (Savari and Gharechaee, 2020; Zobeidi et al., 2022a,b). For instance, according to a study on environmental behaviors, higher levels of education were associated with more positive attitudes toward environmental conservation and a higher likelihood of engaging in environmentally friendly behaviors (Kiatkawsin and Han, 2017). Among the dimensions of environmental value attitudes, those with a biosphere perspective were more likely to endorse rangeland conservation than those with other dimensions. This result is consistent with studies (Chen, 2015; Yeboah and Kaplowitz, 2016; Kiatkawsin and Han, 2017) and confirmed the research hypothesis (1). This can be explained by the fact that people with a biospheric perspective are concerned not only about other people, but also about the environment and other living organisms, and always strive to affect them negatively as little as possible (Yeboah and Kaplowitz, 2016). Therefore, pastoralists with a biospheric perspective are more attracted to RCB and have a positive attitude toward their relationship with the natural environment. Also, they believe that conserving rangeland should be prioritized for ranchers so that they can use the rangeland for the long term (Karimi and Saghaleini, 2021). Livestock owners who have a biospheric value perspective are long-term oriented and reject immediate benefits in the natural environment that could lead to environmental degradation. The second variable of environmental value attitudes associated with rangeland conservation behavior was altruism. This result was consistent with studies (Stern, 2000; Cho et al., 2013; Lind et al., 2015; Valizadeh et al., 2016). Researchers have found that value attitudes influence people’s environmental beliefs and that people with altruistic beliefs permanently attempt not to harm others (Cho et al., 2013). They constantly try to consider the welfare of others (Stern, 2000). Thus, ranchers with an altruistic value attitude always strive not to adversely affect the welfare of other ranchers through their behavior, as they take into account the interests of other ranchers and try to act in an environmentally conscious manner. In addition, these people think about intergenerational equity and believe that rangelands belong to the next generation, so they avoid destructive behavior when using rangelands. A third variable that affects pasture conservation beliefs was egoistic value attitude, which had both negative and significant effects in this regard. This result confirmed the research hypothesis (3). Similarly, studies (Chen, 2015; Fornara et al., 2016; Valizadeh et al., 2016; Veisi et al., 2020) have also found that the egoistic value attitude negatively affect the environment. A person with this value perspective always prioritizes their personal interests over the preservation of the natural environment and other people (Veisi et al., 2020). Egoistic people do not adhere to an environmental ethic or behavior, always highlight their benefits first (Zhang et al., 2020), and act destructively in the natural world. In other words, they pose a constant threat to the environment (Hiratsuka et al., 2018). Therefore, ranchers with an egoistic perspective are those who use RCB less and constantly disturb the balance of grazing land and livestock in the environment. This leads them to increase their livestock numbers to increase profit. These ranchers are the biggest threat to rangelands. They need to increase their awareness and sense of responsibility. Researchers have found that by strengthening the sense of responsibility of the profiteers, people can interact more ethically and expect environmentally friendly behavior (Chen, 2015). In addition, by increasing people’s awareness of the impact of their harmful activities on the environment, their destructive activity in the environment can be reduced (Derckx, 2015).

Moreover, the results showed that individuals’ beliefs could explain 52.8% of their moral norms. Thus, hypothesis (4) of the study was confirmed. Studies (Stern, 2000; Chen, 2015; Lind et al., 2015; Fornara et al., 2016; Zhang et al., 2020) support the findings of this section. Beliefs about environmental behavior are associated with a commitment to the use of natural resources, which can have a positive effect on environmental behavior (Savari et al., 2021). According to Stern (2000), to reduce environmental damage and prevent environmental degradation, it is necessary to strengthen people’s beliefs about environmental conservation, because beliefs can always affect moral norms and sense of responsibility. Ranchers who believe in rangeland conservation, therefore, behave more responsibly in this regard. Finally, the results of the test of hypothesis (5) showed that moral norms influence farmers’ RCB and can explain 53.9% of RCB. Our result accords with (Chen, 2015; Shin and Hancer, 2016; Valizadeh et al., 2016; Veisi et al., 2020). A possible explanation for these results could be that ranchers’ adherence to moral principles is a good predictor of their protective behavior. Since farmers’ behavior is subject to their internal norms, although it is influenced by several complex factors, it is largely determined by the observance of moral principles (Savari and Gharechaee, 2020). The activation of personal norms occurs when the consequences of one’s behavior lead to the destruction of the environment and the individual feels obligated to take responsibility for the natural environment (Stern, 2000). In a moral decision-making situation, people are aware that their actions affect the health of others. Therefore, they feel responsible for their actions and their consequences (Klöckner, 2013). In explaining and predicting behaviors whose consequences go beyond the individual level, such as environmental behaviors, moral norms play an important role (Mullan et al., 2015) and they are one of the most important predictors of environmental behaviors (Shin and Hancer, 2016).

### Policy implication

In this stage, it can be claimed that ranchers who feel morally obligated to care for the range will exhibit the most sustainable behaviors in the natural environment because they feel responsible for the actions and consequences of their behavior and always attempt to not let their behavior lead to negative consequences for the range. If these people receive specific training, they can be used as nature helpers to promote safe behaviors in the environment. In addition, policymakers are advised to provide ranchers with alternative sources of income so that they will not be heavily dependent on grazing lands. This is because people with egoistic value attitudes are too dangerous to rangelands to avoid the overuse of livestock by engaging in other activities. The development of rangeland conservation cooperatives is another important rangeland conservation strategy. The establishment of organizations can undoubtedly develop norms for rangeland conservation among ranchers. In the study area, which covers more than 1000 km^2^, rangeland cooperative is not active and policymakers did not pay attention to it. Another important solution in this area may be the development of grazing licenses for ranchers, which must specify the number of domesticated animals with grazing capacity and monitor them to prevent excessive expansion of livestock.

## Conclusion and limitations of the research

The results of this study revealed that VBN theory was an effective strategy to explain RCB among ranchers in western Iran, which clarified more than 50% of rangeland conservation behavior. This theory reflected the significant influence of psychological models in explaining pastoralists’ behaviors, which have not received much attention in previous research. Consequently, policymakers need to consider these factors when making appropriate decisions. Finally, several important limitations need to be considered. First, some of the variances have not yet been explained. Although psychological models explained a significant portion of the RCB variance, further research may identify other cognitive-behavioral factors. Second, only the VBN theory was examined in this study. For future research in this area, it is recommended that other major psychological theories be examined to identify the most important theories. A third limitation was the overemphasis on psychological factors. It is suggested that future research should also consider other economic and social factors to develop comprehensive and important rangeland conservation planning by ranchers.

## Data availability statement

The original contributions presented in this study are included in this article/supplementary material, further inquiries can be directed to the corresponding author.

## Author contributions

The author confirms being the sole contributor of this work and has approved it for publication.
